# Essential Oils in the Treatment of Various Types of Acne—A Review

**DOI:** 10.3390/plants12010090

**Published:** 2022-12-24

**Authors:** Renata Nurzyńska-Wierdak, Dominika Pietrasik, Magdalena Walasek-Janusz

**Affiliations:** Department of Vegetable and Herb Crops, Faculty of Horticulture and Landscape Architecture, University of Life Sciences in Lublin, 50a Doświadczalna Str., 20–280 Lublin, Poland

**Keywords:** skin diseases, *Cutibacterium acnes*, complementary and alternative medicines (CAMs), antimicrobial and anti-inflammatory activity, synergism, terpenes

## Abstract

Acne is a chronic, common disease that poses a significant therapeutic, psychological and social problem. The etiopathogenesis of this disease is not fully understood. Drugs used in general and external therapy should have anti-seborrhoeic, anticomadogenic, bactericidal, bacteriostatic, and anti-inflammatory properties. Acne treatment is often associated with the long-term use of antibiotics, contributing to the global antibiotic resistance crisis. In order to solve this problem, attention has been paid to essential oils and their terpene components with potent antimicrobial, anti-inflammatory, and antioxidant properties. Research shows that certain essential oils effectively reduce inflammatory acne lesions through mechanisms related to the sebaceous glands, colonization of *Cutibacterium acnes,* and reactive oxygen species (ROS). An example is tea tree oil (TTO), a more commonly used topical agent for treating acne. TTO has antimicrobial and anti-inflammatory activity. The paper presents the latest scientific information on the activity and potential use of specific essential oils in treating acne. Evidence of antibacterial, anti-inflammatory, and antioxidant activity of several essential oils and their main components was presented, indicating the possibility of using them in the treatment of acne.

## 1. Introduction

Skin diseases are a significant therapeutic, psychological and social problem nowadays. The importance of these conditions is regularly underestimated due to their chronic nature and low mortality, but their frequency can be very high, with estimates ranging from 21% to 87% of the population suffering from some skin disease. They account for a quarter of consultations in primary care due to the significant physical and mental impairments associated with it [1]. The most common skin disease is *Acne vulgaris*, which affects 80% of the teenage population; it is even believed that 100% of the population suffered from a more or less severe form of this disease at various times in their lives [2]. Acne is a chronic inflammatory disease, and its pathogenesis is very complex and multifactorial; there are no strict guidelines that would define specific reasons for the occurrence of this skin disease. The development of acne is mainly due to increased sebum production, increased proliferation and decreased shedding of keratinocytes, inflammation, and hyper-colonization of *Cutibacterium acnes* (formerly *Propionibacterium acnes*) [3,4]. In addition to increased sebum production, modification of its composition is also involved in the pathogenesis of acne. The unfavorable change consists of a decrease in the ratio of nutritional lipids palmitic acid C16:0/ palmitoleic acid C16:1 and an increase in the content of linoleic acid (C18:2), which affects the regulation of monocyte differentiation and secretion of cytokines, which contributes to the formation of inflammatory foci [5]. The basic types of acne lesions are inflammatory (papules, pustules, nodules, and cysts) and non-inflammatory (seborrhoea and open or closed comedones); in some cases, secondary inflammatory changes may occur, which may lead to scarring and discoloration. Although acne is not a life-threatening disease, it can have profound psychosocial consequences, resulting in low self-esteem, social isolation, and depression [6]. The treatment of acne is selected based on the severity of acne lesions and the severity of the disease. Usually, acne is classified according to severity as mild, moderate, and severe (fulminant). The best effectiveness in treating acne is obtained with combination therapy aimed at various pathogenetic mechanisms [7]. The most commonly used substances include retinoids, tetracycline antibiotics (e.g., doxycycline, minocycline), clindamycin, benzoyl peroxide, cationic antimicrobial peptide (omiganan), and azelaic acid. Clinical improvement may take 8 to 12 weeks from initiation of treatment, so people affected by acne must be patient and follow the doctor’s instructions [3,4,5]. One form of acne is rosacea, a common, chronic inflammatory dermatosis of the face that occurs predominantly in fair-skinned women in the Northeastern population. The disease is accompanied by intense redness and erythema, many lamp and pustules, as well as telangiectasias of capillary, and secondary changes such as itching, dry skin, burning, or stinging may also occur [8,9]. The process of rosacea development is influenced by external factors such as stress, UV radiation, excessive physical effort, improper diet, stimulants, and internal factors, which include primarily genetic predisposition [10]. In the pathogenesis of rosacea, infections with *Demodex folliculorum* and lipophilic yeast (*Malassezia fur fur*) are also mentioned [11,12].

Due to its location and chronic course, acne can lead to psychosocial disorders. It is crucial to start treatment quickly and take proper care of the skin. Acne treatment therapy is usually selected individually, considering the severity of the disease and its clinical picture, as well as the consequences related to the aesthetic appearance of the skin after its completion. Drugs used in general and external therapy should have anti-seborrhoeic, anti-blackhead, bactericidal, bacteriostatic, and anti-inflammatory properties. Increasing antibiotic resistance of dermal-epidermal acne-causing strains is very difficult to combat, and available synthetic drugs from the group of antibiotics and antimicrobials are becoming less and less effective. The use of retinoids in most cases is complicated by irritant dermatitis, especially after sun exposure, which limits their use in hot seasons [13]. Complementary and alternative medicines (CAMs) are one of the world’s most widespread sources of medicines. Among CAMs, essential oils are the most popular choice for treating many skin conditions due to their strong antimicrobial properties. Resistant strains such as *Pseudomonas aeruginosa* MRSA and *Staphylococcus epidermidis* MRSE have recently become problematic microorganisms due to their resistance to antimicrobial agents [14]. A combination of methods seems to be an adequate solution in treating acne, which in most cases allows for the best therapeutic effects. Anti-acne preparations use, among others, essential oils with an antiseptic effect (tea tree oil, lemon, or petitgrain oil), as well as antibacterial and anti-inflammatory oils that prevent plugging of the sebaceous glands of the skin (bergamot, basil, lavender, thyme oil). Lamlertthon et al. [15] showed the inhibition of the growth of *C. acnes* by 19 essential oils, among which the most potent antibacterial activity was found in the oils of *Citrus hystrix* DC., *Cymbopogon citratus* (DC.) Stapf, *Syzygium aromaticum* (L.) Merr. & Perry and *Michelia alba* DC. Daud et al. [16] showed the antibacterial effect of cinnamon, tea tree, and rosemary oils on *C. acnes* and *S. epidermidis*, considered the main skin bacteria contributing to the formation of acne, evaluating the tested oils as effective anti-acne agents. The following part of the paper presents the results of research on the chemical composition and therapeutic effect of selected essential oils: tea tree, eucalyptus, myrtle, lavender, oregano, thyme, and lemon, related to the effect on acne skin and the potential possibilities of using these oils in the treatment of various forms of acne.

## 2. Essential Oils with Potential Anti-Acne Effects

### 2.1. Tea Tree Essential Oil

Tea tree oil (TTO) is obtained by steam distillation of the leaves and twigs of the native Australian tree *Melaleuca alternifolia* Maiden & Betche of the Myrtaceae family. In Australia, it has been used for a long time for therapeutic purposes, cleaning and treating wounds, against colds, and headaches, for oral hygiene and inhalation, as an anti-scorbutic agent, and for the preparation of disinfectants [17]. TTO is characterized by high bioactivity and is an effective bactericidal, fungicide, and antiseptic agent, relatively safe and effective, with significant potential for use in the health and cosmetics industry. TTO is often sold as a pure oil or a component of ready-made antiseptic and antibacterial products (soaps, creams, kinds of toothpaste, mouthwashes, balms, and acne serums) [18,19]. TTO is a colorless or light yellow, multi-component liquid with a characteristic peppery-spicy aroma, sometimes reminiscent of camphor. It also has a cooling effect similar to menthol but less intense [18]. The oil contains about 100 components present in various concentrations, among which the following predominate: terpenen-4-ol, γ-terpinene, α-pinene, and α-terpinene (Table 1).

TTO has a broad spectrum of antimicrobial activity, showing effects in treating acne [26,27]. It is usually used in various topical preparations. It contains over 80–90% of monoterpenes, including terpinen-4-ol, p-cymene, α-terpinene, 1,8-cineole, limonene, α-terpineol, terpinolene, sabinene and α-pinene [20,28,29]. Furthermore, it exhibits antioxidant, anti-inflammatory, analgesic, and anticancer activity [20]. Such numerous and valuable properties make it very popular. It supports the therapy of many skin diseases and their appendages, such as acne vulgaris, dandruff, dandruff versicolor, psoriasis, seborrhoeic dermatitis, diaper rash and eczema, corns and hardening, foot and scalp mycoses, nail yeast infections, diabetic ulcers and varicose veins, burns, boils, and paronychia. TTO is considered one of the most potent natural antiseptics. Antibacterial (*S. epidermidis*, *C. acnes*), antifungal (*Candida albicans*, *Trichophyton mentagrophytes*, *Microsporum canis*, *M. gypseum*), anti-inflammatory and antioxidant effects of tea tree oil have been demonstrated [28]. Lee et al. [30] showed that TTO and its components, terpinen-4-ol, α -terpinolene, α -terpinene, and α -terpineol, had strong inhibitory activities against *C. acnes* and *S. aureus*. The authors note that while terpinen-4-ol is the primary active ingredient responsible for TTO’s antimicrobial effectiveness, minor TTO components also contributed to its effectiveness. The strong antibacterial effect of tea tree oil is obtained even at low concentrations. It has been proven that in the concentration range of 0.6–30 mg/mL, it inhibits the development of Gram-positive aerobic cocci of *S. epidermidis*, and in the concentration of 1–5 mg/mL, it has a bactericidal effect against 32 strains of *C. acnes* bacteria, which were isolated from acne lesions [26,31]. TTO significantly decreased acne formation by inhibiting the growth of acne-related bacteria, *C. acnes* and *S. aureus*, and reducing acne-caused inflammation. TTO concentration of less than 5% is more suitable and safer for treating acne than higher concentrations [30].

Using the oil on the external parts of the body is safe, and side effects are rare. Skin side effects, which usually occur only in people with sensitive or allergic skin, can be reduced using low concentrations of the preparation. TTO is applied to the skin within 30 min. and absorbed rapidly. It can migrate to the dermis (cutis verai) and get into the bloodstream, lymphatic system, and skin nerve endings. Applied topically while penetrating the skin layers, it does not change its chemical composition, thus retaining its original healing properties [17,27]. The lipophilic components of the oil can also quickly get inside the cells of microorganisms, causing inhibition of metabolism and their death [32]. TTO has great potential in treating acne due to its antimicrobial effects, ability to reduce scars, and ability to promote wound healing. In addition, it offers advantages over antibiotics due to its low impact on the development of antimicrobial resistance and susceptibility [33]. A broad spectrum of antibacterial and anti-inflammatory properties is critical in treating acne; hence TTO may be a valuable therapeutic ingredient. Mazzarello et al. [34] proved that a cream containing TTO 3%, propolis 20%, and *Aloe vera* 10% has antibacterial and anti-inflammatory properties and is more effective in reducing acne, erythematous scars, acne severity index, and the total number of lesions compared to the preparation of synthetic origin, e.g., erythromycin. Azelaic acid (AzA) poses some application problems in treating acne due to poor water solubility, low skin permeability, and dose-dependent side effects. Bisht et al. [35] proposed a synergistic combination of AzA with TTO in the form of a hydrogel composite based on microemulsion, assessing it as effective and safe in in vitro, in vivo, and ex vivo tests. The formulations developed protected the direct exposure of the drug to the skin, thereby reducing side effects and showing the best penetration and retention characteristics in the skin compared to formulations available on the market. The in vitro antibacterial efficacy of the products revealed an improved zone of inhibition and low MIC values against *Staphylococcus aureus*, *S. epidermidis* and *C. acne*. The above positive reports allow the use of TTO as an alternative therapy for treating various forms of acne.

### 2.2. Eucalyptus Essential Oil

*Eucalyptus globulus* Labill. is one of the most widely used medicinal plants in the world. The species comes from Australia but is now cultivated in many countries due to its ease of adaptation and cultivation, tolerance to a wide range of environmental conditions, and rapid growth. There are about 900 different species of eucalyptus, of which only about 30% contain an essential oil that can be harvested on a larger scale. The above-ground parts of eucalyptus have been used for centuries as a traditional medicine for various health problems, such as respiratory infections, toothache, diarrhea, and stomatitis [36,37]. *E. globulus* oil (EGO) is distilled from the leaves with a yield of 2.6% [37]. The oil composition shows significant differences depending, e.g., on the time of harvest, geographical region, method of cultivation, or distillation (Table 2). The essential components of EGO include 1,8-cineole (eucalyptol), α-pinene, globulol, terpinen-4-ol, β-phellandrene, caryophyllene, α-terpinyl acetate, limonene, aromadendrene [38,39,40,41]. Referring to the British and European Pharmacopoeia, oil is a pharmaceutical grade if it contains at least 70% 1,8-cineole [42]. The content of 1,8-cineole in EGO from different parts of the world can be very high: 83.9–90.0% (Brazilian oil from São Paulo state), 85.8% (Brazilian oil from Minas Gerais state), 86.5% (Indonesia), 85.8% (Montenegro), 81.9% (India), 90.0% (Australia), 95.5% (Italy) and 98.9% (Argentina) [36].

Recent studies have highlighted the antibacterial, antifungal, analgesic, and even anticancer properties of eucalyptus leaf extracts and essential oil, which are associated with the reported anti-inflammatory and antioxidant properties [46]. EGO, by inhibiting the production of tyrosinase and melanin, has a depigmenting and even skin tone effect, effectively reducing acne scars [41]. Research conducted by Bhatt et al. [47] determined the range of biocidal activity against various bacterial strains using eucalyptus oil, showing the inhibitory effect of EGO on some pathogenic bacteria associated with the development of acne. It has also been found that the oil is very effective in reducing the size of enlarged sebaceous glands and thus controlling the production of sebum, the excessive amount of which promotes the formation of acne lesions. The EGO has been proven to have a dual effect, reducing sebum production and controlling the secondary infectious stage by other microorganisms, establishing an alternative pathway for acne management [47]. Göger et al. [45] suggest that the EGO and its major constituent, 1,8-cineole works against skin pathogenic bacteria and show an anti-inflammatory effect. The authors confirmed the inhibitory effect of EGO on the activity of *C. acnes*, *S. aureus,* and *S. epidermidis* (80.2% 1,8-cineole), especially against humans pathogen *S. epidermidis*. 1,8-cineole was found to be much more effective against *S. epidermidis* compared to other tested strains. The research of Athikomkulchai et al. [48] shows that EGO has antimicrobial activity against *C. acnes*. An oil-in-water cream containing 2% eucalyptus oil was more effective than a commercial anti-acne gel with 5% benzoyl peroxide [47]. Bey-Ould Si Said et al. [49] confirmed the antibacterial and bactericidal effect of essential oil from *E. globulus* fruit on reference pathogenic strains: *Bacillus subtilis*, *Listeria innocua*, *Escherichia coli*, and *Pseudomonas aeruginosa*. Interesting information was provided by Assaggaf et al. [50], describing the synergism of EGO with honey. It was proven that this combination enhanced the anti-inflammatory, antioxidant, and dermatoprotective effects and could inhibit lipid peroxidation, possibly due to the synergistic effect [51]. Due to its confirmed anti-inflammatory and antiseptic effect against bacteria involved in the pathogenesis of acne, EO is one of the alternative forms of support in the fight against bacterial or inflammatory skin diseases.

### 2.3. Myrtle Essential Oil

Myrtle (*Myrtus communis* Linn.) is a well-known medicinal plant from the Myrtaceae family. It is an aromatic evergreen shrub or small tree which is 1.8–2.4 m tall, native to Southern Europe, North Africa, and Western Asia. Myrtle leaves are dark green, shiny, glabrous, leathery, and ovate to lanceolate with a rigid, aromatic structure. Myrtle blooms during summer; the flowers exude a sweet, aromatic fragrance. The fruit is a berry, initially pale green, becoming dark red as it matures, and later blue-black. Unripe fruits are bitter, and ripe fruits become sweet [51]. *M. communis* is an oil plant; the essential oil is isolated from various organs, leaves, flowers, and fruits. The oil obtained from the leaves is of industrial importance due to its pleasant smell (perfume) and biological effect (aromatherapy). The essential oil obtained from myrtle leaves (MCLO) chemically belongs to two basic types: *cineoliferum* type, rich in terpenes (α-pinene, limonene) and terpenoid oxides (1,8-cineole), and the *myrtenilacetatiferum* type, rich in terpene esters (terpenyl acetate, linalyl acetate, bornyl acetate) and terpenoid oxides (1,8-cineole) [52]. The quantity and quality of MCLO depend on geographical origin and harvest time, but genotype may also play a role in the chemical variability (Table 3). The oil from the leaves harvested in March contained more α-pinene and linalool and less 1,8-cineole than in October [53]. MCLO of plants growing in various regions of Iran contains mainly: α-pinene, 1,8-cineole, linalool, and α-terpineol, with eugenol, δ-3-carene, 1,8-cineole, and α-terpineol [54]. The oil from the leaves of the Tunisian and Algerian populations of *M. communis* turned out to be rich in monoterpene hydrocarbons (53.38%), especially α-pinene and α-limonene. The share of individual components varied within and between populations: the highest share of α-pinene (45.4%) and 1,8-cineole (35.7%) were found in the Algerian population, and α-limonene in the Tunisian population (18.16%) [55]. MCLO has a yellowish color and a very aromatic fragrance; the extraction yield from the dry material is in the range of 0.7–1.3% [56,57]. The essential oil content of myrtle fruit is relatively low compared to the yield obtained from this plant’s leaves or flowers. The fruit oil is obtained with a yield of 0.10–0.59% [53,58] and contains mainly geranyl acetate, a compound with a floral or fruity rose aroma, as well as: 1,8-cineole, α-terpinyl acetate, methyleugenol, linalool, α-terpineol, β-caryophyllene, α-humulene, trans-caryophyllene oxide and humulene II epoxide [58]. Giuliani et al. [53] showed an almost twice as high content of α-pinene and 1,8-cineole and a smaller amount of α-terpineol in the oil obtained from fruits harvested in October than in July.

Myrtle is a promising source of alternative antimicrobial agents against the growing number of pathogenic microbes resistant to conventional antibiotics and antioxidants. Myrtle oil, primarily obtained from the leaves, has also shown good anti-diabetic and anti-inflammatory effects [57,63]. MCLO has antimicrobial (antibacterial, antifungal, and antiviral) and antioxidant properties [64,65]. The 10 μL of the oil significantly inhibited the growth of five tested bacteria, *E. coli*, *S. aureus*, *B. subtilis*, *Salmonella* sp., and *Listeria* sp. [55]. Hartiti et al. [56] reported that MCLO showed moderate inhibitory activity against *S. aureus*, *Acinetobacter baumannii*, *Klebsiella pneumoniae*, and *S. epidermis*, and *P. aeruginosa* was found to be very resistant to the oil. Pinene is the most widely found monoterpenoids, which exhibits various biological activities [66]. The α-pinene, one of the main components of MCLO, was found to be active against *S. aureus*, *S. edipermidis*, and *C. acnes* [67]. Mahmoudvand et al. [62] showed a strong antileishmanial effect of MCLO, indicating it is a potential source of production of new agents against cutaneous leishmaniasis. This infection causes chronic skin lesions and leaves permanent scars with deformation of the infected area. A study by Kim et al. [68] has clinically proven that MCLO has the effect of convergence, reduction of erythema, removal of sebum and dead skin cells, and antibacterial effect on the facial skin of Korean women. These studies show that MCLO is a safe, skin-soothing substance that effectively treats acne. Further results were provided by Mazzarello et al. [34], comparing a commercial product (Acnatac gel) based on clindamycin-tretinoin (CTG) with a galenic product containing two essential oils (myrtle and oregano oil) and tretinoin (MOTC) to evaluate its anti-acne properties and effects on the skin. The authors chose a product based on clindamycin because it has recently replaced erythromycin, which is increasingly showing the occurrence of bacterial resistance. Essential oils were selected for their antibacterial and anti-inflammatory properties. The obtained results indicate that MOTC, compared to CTG, has the same anti-acne effectiveness and increased anti-inflammatory activity; MOTC showed, compared to CTG, anti-acne, and anti-inflammatory effects, thanks to essential oils able to reduce erythema in vivo and retinoid-induced lesions.

### 2.4. Lavender Essential Oil

Lavender (*Lavandula angustifolia* Mill.), a shrub from the Lamiaceae family found on the shores of the Mediterranean Sea, is valued primarily for its pleasant aroma [69]. *L. angustifolia* flowers are used to obtain valuable lavender oil (LAO) by distillation. The most frequently identified compounds in the LAO are linalool, linalyl acetate, 1,8-cineole, camphor, β-caryophyllene, borneol, cis-β-ocimene, lavandulyl acetate, terpinen-4-ol, α-terpineol, β-farnesene [70]. The composition of the LAO shows high variability, which is influenced by many factors (Table 4). Among them, the genotype, cultivation conditions, harvest date, type of raw material, drying, and distillation methods are considered the most important [69,70,71,72,73,74,75,76,77]. In the Egyptian LAO, higher amounts of δ-carene were found (up to 17.4%), the oil of plants from Italy had a higher content of α-bisabolol (6.75–11.87%), and plants cultivated in Poland had a higher amount of geraniol (5.3%) [70]. The composition of LAO determines its usefulness in pharmaceutical or cosmetic production. The oil containing significant amounts of linalool, linalyl acetate, and small amounts of camphor is used in the perfume industry [78,79]. The LAO is one of the most valuable oils used in cosmetology, pharmacy, medicine, and aromatherapy due to its strong antibacterial and antifungal properties [70,75,80]. The antibacterial properties of lavender oil result primarily from the high content of linalool and linalyl acetate [81].

Antibacterial and antifungal properties have made LAO one of the most commonly used oils on the skin’s surface in the treatment of acne, eczema and psoriasis; it also improves skin condition [83]. The advantage of using the LAO is that it is often applied undiluted to the skin; the skin quickly absorbs the oil and its ingredients. After topical application combined with massage, linalool and linalyl acetate were detectable in plasma at maximum levels after about 19 min [84]. Lavender oil has a bactericidal effect, even on some antibiotic-resistant microorganisms, which is essential in the case of long-term acne treatment [85,86]. The effectiveness of LAO depends on its chemical composition. In studies using commercial lavender oil (produced by Etja) and oil obtained from plants from the Crimean Peninsula on mixed skin microflora, it was shown that both tested oils affected the bacilli of both Gram-positive and Gram-negative bacteria but did not inhibit the growth of Gram-positive cocci. In addition, both oils reduced the number of mixed facial skin microflora cells, but Etja oil was more efficient [87]. On the other hand, studies conducted using commercial lavender oil against *C. acnes* did not show a practical bactericidal effect [88], and in other research [89], the effect of the oil was low (MIC values 4 mg/mL). It can be assumed that the activity of LAO depends, among others, on its chemical profile, including the share of linalool. Previous studies have shown that this compound has antioxidant, anti-inflammatory, anticancer, cardioprotective, and antimicrobial properties and also against opportunistic bacteria (*P. aeruginosa* and *S. epidermidis*) [90]. Linalool associated with standard antibiotics may increase antibacterial effectiveness, resulting in synergistic activity against bacterial strains of clinical importance, which makes it possible to act on resistant strains [91]. Adaszyńska-Skwirzyńska et al. [92] demonstrated a synergistic effect between LAO and gentamicin and between linalool and gentamicin against *S. aureus* ATCC 25923 and *S. aureus* MRSA. Linalool interferes with the morphological switch and biofilm formation of *C. albicans*. This compound exhibited antifungal activity against *C. albicans* with a minimum inhibitory concentration (MIC) of 8 mM, and sub-MIC concentrations of linalool also inhibited the formation of germ tubes and biofilms in that strain [93]. Lavender oil is recommended for prophylaxis and topical treatment of superficial infections but is not recommended for use in treating deep (deep-seated) infections [94]. Kim and Shin [95] demonstrated in vivo studies that a mixture of TTO (3%) and LAO (2%) applied topically for 4 weeks significantly reduced both the total population of *C. acnes* and the number of inflammatory lesions. These studies confirm that the tested essential oils have antimicrobial activity and improve acne lesions in vivo, and LAO can be used as an alternative treatment method for patients unwilling or unable to use antibiotics to treat acne.

### 2.5. Oregano Essential Oil

*Origanum vulgare* L. from the Lamiaceae family is an aromatic plant commonly known in the Mediterranean Basin, Central, and Northern Europe, North America, and Asia. Oregano has long been used as a medicinal herb in ethnopharmacological preparations for treating various diseases, such as upper respiratory tract infections, indigestion, painful menstruation, rheumatoid arthritis, and diseases related to the urinary tract. Among the numerous varieties of oregano, the Greek oregano (*O. vulgare* ssp. *hirtum*) deserves special attention, which is considered exceptionally valuable due to its excellent quality and high concentration of essential oil [96,97]. The high content of essential oil, the concentration of which, depending on the origin, may reach 8.2%, is responsible for the therapeutic effect and the characteristic aroma and taste of oregano [98]. The oil content in the herb of European plants *O. vulgare* ranges from 0.03% to 4.6% [99]. The essential oil of *O. vulgare* (OVO) accumulates mainly in leaves and flowers, and its chemical composition varies (Table 5). The main OVO fractions include acyclic and cyclic monoterpenes (1,8-cineol, γ-terpinene, linalool, geraniol, β-myrcene, trans-sabinene, α-pinene, β-citronellol, and terpinen-4-ol), sesquiterpenes (β-caryophyllene, germacrene-D) and aromatic hydrocarbons (p-cymene) [100,101]. Various monoterpene chemotypes have been described, which have been defined based on the content of the main compounds [102]. Based on many analyses, three basic chemotypes of *O. vulgare* were determined, depending on the ratio of acyclic linalool/linalyl acetate, cymyl-compounds, and sabinyl-compounds. Cymyl and acyclic chemotypes are usually found in plants from the Mediterranean climate, while the sabinyl chemotype is characteristic of plants from continental climates [99].

OVO has found application in the treatment of some skin diseases. The oil’s potent antioxidant, antimicrobial, and anti-inflammatory effects are closely related to the anti-acne, regenerating, and anti-aging properties. Antioxidant properties are attributed to carvacrol, thymol, and p-cymene, which can form chemical complexes with metal ions and free radicals [101]. Research by Nostro et al. [103] proved that OVO and its components, thymol and carvacrol, effectively inhibited the activity of methicillin-resistant *S. aureus* and *S. epidermidis* bacteria. The best MIC values were shown by carvacrol (0.015–0.03%), followed by thymol (0.03–0.06%). Recently, the focus has been on developing modern preparations that would be biocompatible with human skin. Bora et al. [104] proved that nanosystems loaded with OVO can be a natural, alternative treatment for skin ailments, including acne, irritation, wounds, or skin aging. Taleb et al. [100] proved the strong antibacterial effect of OVO against *C. acnes* and *S. epidermidis* and the anti-acne potential of topical OVO nanoemulsions in vivo in an acne-induced mouse model to overcome the limitations of topical anti-acne antibiotics. The use of the preparation contributed to the reduction of inflammation and better healing of tissues. The preparation showed a better therapeutic and antimicrobial effect than the reference antibiotic. Similarly, Mazzarello et al. [34] showed a superior effect of a galenic preparation containing *O. vulgare* L. and *M. communis* L. essential oils in mild to moderate acne compared to a commercial product containing clindamycin and tretinoin. The preparation with the addition of oils showed a better effect in reducing papular erythema and soothing irritation caused by retinoids. Avola et al. [105] showed that OVO contributed to reducing some parameters related to inflammation and supported the motility of keratinocytes during wound healing. In summary, OVO is a promising source of bioactive ingredients. Due to its anti-inflammatory, antioxidant, and antibacterial properties, it may be an interesting component of preparations in treating acne and other skin diseases accompanied by inflammation.

### 2.6. Thyme Essential Oil

Thyme (*Thymus vulgaris* L.) from the Lamiaceae family is an aromatic shrub growing up to 30 cm in height from the Mediterranean. Thyme leaves are ovate-lanceolate, hairy on both sides, and the flowers are pink, rarely whitish; these organs abound in essential oil. The species presents a large variety of forms and chemotypes. As a result of the variability of chemotypes, the aroma of thyme can be unique, even resembling the smell of lemon or verbena [106]. The oil is isolated from thyme herb with a yield of 1.25% [107]. The main components of *T. vulgaris* oil (TVO) include thymol, p-cymene, 1,8-cineole, γ-terpinene, and carvacrol [107,108,109] (Table 6).

TVO has antiseptic, antimicrobial, astringent, anthelmintic, healing, tonic, carminative, antiseptic, antiviral, and anti-inflammatory effects [107,114]. It exhibits intense antibacterial activity against many pathogenic bacteria and can be used as an alternative antibacterial and antioxidant agent [110]. Studies by Boskovic et al. [108] proved a broad antibacterial activity of TVO against *Salmonella enteritidis*, *S. thyphimurium*, *S. aureus*, methicillin-resistant *S. aureus*, *E. coli*, and *B. cereus*. Dauqan and Abdullah [112] confirmed the antimicrobial activity of TVO and thymol in vitro against *E. coli* strains. Thymol and carvacrol are the most active ingredients with antimicrobial activity [113]. Fachini-Queiroz et al. [115] report that TVO and carvacrol have anti-inflammatory effects, which can be attributed to the inhibition of inflammatory oedema and leukocyte migration. Thymol does not reduce the formation of oedema but causes irritation, possibly mediated by the release of histamine and prostanoids. Thymol is active against enterobacteria and cocci bacteria [114]; however, crude essential oil is the most effective. Ahmad et al. [113] investigated the antimicrobial interactions between TVO components, determining the contribution of less active ingredients to enhancing the oil’s antimicrobial activity. In a combination study, interactions were 21% synergistic, 42% additive, 36% neutral, and 1% antagonistic. The most pronounced synergistic effect was observed between the weakly active p-cymene and the highly active carvacrol against *Moraxella cattarhalis*. Of the 147 tested combinations, only two antagonistic effects were noted, which concerned the combination of thymol and borneol against *M. cattarhalis* and the combination of γ-terpinene and carvacrol against *S. aureus*. When thymol (a more potent antimicrobial) was combined with the weaker p-cymene, synergistic interactions were shown against four pathogens, and the combination of carvacrol and p-cymene showed synergy against five of the seven pathogens tested. The results of the above studies show that compounds with high activity together with compounds with lower activity have an additive and even synergistic effect and increase the antimicrobial effectiveness of TVO.

Melo et al. [116], evaluating the antibacterial activity of 19 essential oils against three strains of bacteria associated with acne (*C. acnes*, *S. epidermidis*, and *S. aureus*), selected the most promising oils for topical acne treatment based on minimum inhibitory concentrations and chemical composition: thyme oil, cinnamon, and clove oil. Of the 10 essential oils that Zu et al. [88] studied, thyme and cinnamon oils showed the most potent antibacterial activity against *C. acnes* and cytotoxic activity against human tumor cell lines A549, PC-3, and MCF-7. Studies by Gonçalves et al. [117] indicate that TVO in the preparation had a similar antimicrobial effect as the essential oil itself and effectively inhibited the growth of microorganisms. The authors suggest that the topical application of TVO is a promising alternative to cosmetic and phytotherapeutic applications. Abdelhamed et al. [111] showed a potent antimicrobial and antibiofilm effect of TVO against *C. acnes* and *S. epidermidis*, recognizing the main antimicrobial components as phenols and terpenoids. Thyme oil nanoemulsion had a strong antibacterial and anti-inflammatory effect compared to reference antibiotics, suggesting its effectiveness as a natural alternative in treating acne. Biofilm production is an essential microbial virulence factor that plays a crucial role in their resistance to conventional antibacterial and antifungal agents. Proškovcova et al. [118] showed high efficiency of OVO and TVO in the adherence phase and formation of *C. albicans* biofilm. Cabarkapa et al. [119] showed that *Origanum* and *Thymus* oils and their main components (thymol and carvacrol) are effective against pre-formed 48-h biofilms of *S. enteritidis*, and biofilm reduction occurred over time and in a dose-dependent manner. The results indicate that OVO and TVO are potential agents for antifungal and antibacterial treatment or prophylaxis by reducing pathogen resistance.

### 2.7. Lemon Essential Oil

Lemon—*Citrus limon* (L.) Burm. f. (*C. × limonia, C. limonum*) is one of the best-known and most widely used species of the genus Citrus. It is a tree reaching 2.5–3 m in height with evergreen, lanceolate, elongated leaves and bisexual white flowers appearing singly in the leaf axils. The fruit of the lemon is an oblong, oval, pointed berry of green color, turning yellow during ripening. The pericarp of *C. limon* is made of a thin wax-coated exocarp with an outer mesocarp (flavedo) and an inner, white, spongy mesocarp (albedo). From the fresh outer parts of the pericarp (exocarpium) by distillation or cold pressing, the essential oil is obtained—*C. limon* oil (CLO) (*Citrus limon aetheroleum*, *Limonis aetheroleum*, *Oleum Citri*). Cold-pressed citrus oils cause phototoxic reactions because they contain photoactive furocoumarins (psoralens), including the most phototoxic bergapten and other derivatives, such as bergamottin, citropten, herniarin, or oxypeucedanin [120]. Lemon pericarp contains 0.6–0.8% of colorless or yellow essential oil, which has a characteristic, strong lemon smell [121]. CLO is rich in bioactive monoterpenoids such as D-limonene, β-pinene, and γ-terpinene (Table 7). The content of limonene and citral (a mixture of geranial and neral) in CLO depends on various factors: genotype, place and cultivation conditions, and harvest date [122].

CLO has antimicrobial and anti-inflammatory properties and is used in dermatology, e.g., in treating acne vulgaris and rosacea [14]. The antibacterial activity of CLO is broad; the oil was active against Gram-positive (*B. subtilis*, *S. capitis*, *Micrococcus luteus*) and Gram-negative (*Pseudomonas fluorescens*, *E. coli*) bacteria [127,128]. Liu et al. [129] report that CLO has an inhibitory effect against *Streptococcus mutans* and effectively reduces the adhesion of bacteria to the glass surface. Effectively reduces glucosyltransferase (Gtf) activity and Gtf transcription in a dose-dependent manner. The dominant component of CLO is limonene (Table 7), one of the most common components of essential oils, a precursor of monoterpenoid biosynthesis in plants. The antimicrobial properties of limonene, both as a pure compound and as one of the main constituents of the essential oils of several plant species, have been well studied. Comparing the inhibitory effects of D-limonene and the essential oils of orange, lemon, grapefruit, and tangerine fruits, pure limonene and limonene oils were found to be highly effective as antimicrobial agents [130]. Aliyah et al. [131] showed the essential oils obtained from fresh leaves of *C. medica* L. have antibacterial activity on *S. aureus*, *C. acne*, and *C. albicans*, suggesting that this activity may be due to the presence of limonene and citral compounds. Han et al. [132] showed that limonene effectively inhibited the growth of *S. aureus* ATCC 6538 at a minimum inhibitory concentration (MIC) of 20 mL/L. It was confirmed that limonene caused the destruction of cell morphology and the integrity of the bacterial cell wall, damaging the cell membrane, increasing membrane permeability, and reducing the metabolic activity of the respiratory system. The antibacterial activity of CLO and limonene give vast possibilities for using these products to treat acne. Fitri et al. [133] determined the formula of an anti-acne serum composed of lime oil (lime peel oil) and patchouli oil, and olive oil in a ratio of 11:1:18, proving while the serum and lime oil rich in D-limonene turned out to be significantly more active against *C. acne* than TTO, patchouli oil, and acne gel.

**Figure 1:**
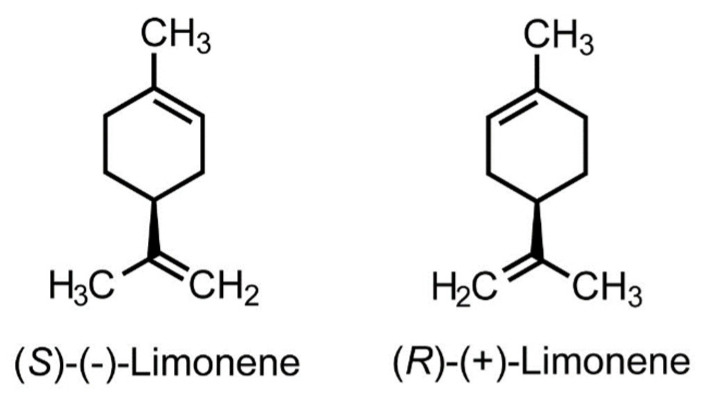
Structure of limonene.

CLO showed anti-inflammatory effects in mice in the formalin assay, reducing cell migration, cytokine production, and carrageenan-induced protein extravasation. These effects were also obtained with the use of pure D-limonene. The anti-inflammatory effect of *C. limon* essential oil is probably due to the high concentration of D-limonene [124]. Limonene is a terpene compound widespread in plants and exists in two enantiomeric forms: R and S (Figure 1). S-(−)-enantiomer, also known as D-limonene, is the main compound in essential oils from *Citrus* spp. peels, R-(+)-limonene (l-limonene) is found in some essential oils and is a cheap by-product of orange cultivation. The D-limonene has a distinct orange smell, and L-limonene has an aroma reminiscent of a combination of pine and turpentine. Limonene is a low-molecular lipophilic compound; therefore, it can quickly saturate cell membranes and thus provide cells with anti-inflammatory protection. Various researchers have verified the anti-inflammatory properties of limonene. Analysis of pure limonene enantiomers showed that (L)-(+)-limonene was about three times less active than the (D)-(−)-limonene enantiomer, and the racemic mixture showed an activity intermediate between the values of the two isomers tested separately [130]. This information helps to explain the differences in the anti-inflammatory activity of different essential oils, as their pharmacological activity may depend on the particular enantiomer, ratio of enantiomers, or both.

## 3. Conclusions

Acne is a chronic inflammatory disease of the hair follicles. The disease has four leading causes: (1) sebaceous hyperplasia and hyperseborrhea, (2) hyperkeratinisation and subsequent keratinocyte accession, (3) colonization of *C. acnes* and *S. albus*, and (4) inflammation and immune response [13]. Inflammation is a complex process essential to the host’s defense system. Excessive production of specific inflammatory mediators can lead to chronic diseases. Dermatitis, which adversely affects its functioning and appearance, also affects the psyche, which is essential in treating chronic skin diseases [134]. Modern therapeutic methods supplemented with effective and safe natural remedies such as essential oils, their ingredients, or both, as well as an appropriate diet, may be a suitable solution covering the entire problem of acne. Essential oils that can be recommended in anti-acne therapy should be distinguished by a strong and documented antibacterial, anti-inflammatory, and antioxidant effect, but also a calming, antidepressant, and toning effect because this disease influences the physical and the mental sphere. The essential oils listed in the review: tea tree, eucalyptus, myrtle, lavender, oregano, thyme, and lemon, meet these requirements and can be recommended as anti-acne agents. In the treatment of acne, some essential oil components, such as linalool, limonenen, thymol, carvacrol, α- and β-pinene, 1,8-cineole, terpinen-4-ol, can also be used. An acne treatment program that uses a combination of tea tree and lavender oils, citrus oils, thyme and/or their components, and other highly active oils can be effective in improving acne lesions due to its antibacterial, antibiofilm, inflammatory, and sebum production effects. The high safety profile of essential oils used according to the recommendations makes it possible to use them in long-term therapy without the risk of side effects. Acne intervention programs that combine essential oil therapy with more conventional treatment may effectively reduce the population of *C. acnes*, *S. epidermidis* and *S. aureus,* and other microbes, destroying biofilm structure and reducing lesions and sebum secretion in the population diagnosed with various forms of acne. CAM refers to forms of health care that are used in addition to (complementary) or instead of (alternative) traditional medical treatment. Complementary therapies are often based on traditional knowledge, but many of them are scientifically validated. Scientific evidence suggests that some essential oils and their components may be helpful in anti-acne therapy. However, much more research and further evidence are needed to fully exploit the therapeutic potential of essential oils.

## Figures and Tables

**Table 1:** The main compounds of *M. alternifolia* leave essential oil.

Compound	Content (%)	References
Terpinen-4-ol	17.3–48.0	[20,21,22,23,24,25]
γ-terpinene	10.8–23.1	[20,21,22,23,24,25]
α-pinene	1.0–21.6	[20,21,22,23,24,25]
α-terpinene	2.6–11.3	[20,21,23,24,25]
Limonene	1.6–9.4	[23,24,25]
α-terpineol	1.5–8.0	[20,21,22,23,24,25]
1,8-cineole	0.0–15.0	[20,21,22,23,24,25]
ρ-cymene	0.5–8.0	[20,21,22,23,24,25]
α-terpinolene	1.5–6.4	[20,21,22,23,24,25]

**Table 2:** The main compounds of *E. globulus* essential oil.

Compound	Content (%)	References
1,8-cineole	11.3–76.6	[37,39,40,41,42,43,44,45]
p-cymene	8.8–20.2	[37,40,41,44,45]
α-pinene	5.6–20.1	[39,40,41,42,43,44,45]
D-limonene	6.2	[41,42,45]
γ-terpinene	8.9	[40,41,44]
α-terpineol acetate	4.8	[40,41,45]
Alloaromadendrene	4.0	[43]

**Table 3:** The main compounds of the oil are distilled from the *M. communis* leaves.

Compound	Content (%)	References
α-pinene	19.4–59.0	[55,56,59,60,61]
1,8-cineole	13.2–61.0	[55,56,59,60,61]
Myrtenyl acetate	8.3–21.3	[56,62]
α-limonene	3.2–19.8	[55,61]
Linalool	1.7–12.7	[59,60]
Linalyl acetate	8.6	[60]
α-terpynyl acetate	4.6	[60]

**Table 4:** The main compounds of the oil distilled from the *L. angustifolia* flowers.

Compound	Content (%)	References
Linalool	8.9–53.4	[70,74,75,76,79]
Linalyl acetate	14.2–56.7	[70,75,76,79]
1,8-cineole	10.15–28.3	[70,75,79]
Camphor	0.5–28	[70,75,79]
Caryophyllene	4.7–24.12	[70,75,79,82]
Borneol	2.0–14.7	[70,75,79,82]
cis-β-ocimene	0.36–3.9	[70,75,79]
Lavandulyl acetate	4.4–8.62	[70,75,79]
Terpinen-4-ol	3.4–10.2	[70,74,75,76,79,82]
α-terpineol	0.32–9.17	[70,79,82]
β-farnesene	0.67–4.5	[70,75,79]

**Table 5:** The main compounds of the *O. vulgare* essential oil.

Compound	Content (%)	References
Carvacrol	3.1–92.9	[96,98,99,101,102]
β-citronellol	72.7–85.3	[102]
Linalool	0.3–84.7	[99,102]
Pulegone	44.3–77.5	[101]
α-terpineol	0.1–52.8	[99,102]
cis-sabinene hydrate	0.3–46.6	[96,98,99]
γ-terpinene	0.2–34.2	[97,98,99,101,102]
Linalyl acetate	0.3–33.0	[98,99]
Caryophyllene oxide	0.1–32.9	[97,98,99,102]
p-cymene	0.1–26.0	[96,98,99,101,102]
β-caryophyllene	0.4–25.1	[98,99,101,102]
Terpinen-4-ol	16.3–24.9	[101,102]
1,8-cineole	1.5–20.8	[99,100,101,102]
Germacrene D	2.4–20.6	[99]
cis-β-terpineol	16.5	[102]
Thymol	0.2–15.9	[96,98,99,101,102]
cis-β-ocimene	0.1–15.6	[99]
α-terpinene	0.1–15.1	[99,102]
Carvacrol methyl ether	0.1–13.7	[98,99]
Sabinene	0.5–12.5	[96,98,99,102]
α-himachalene	12.2	[102]
Humulene	7.7–11.5	[102]
β-pinene	0.3–11.7	[98,102]
Eugenol methyl ether	9.8	[102]
o-cymene	5.9–8.9	[101]

**Table 6:** Prevailing components of *T. vulgaris* essential oil.

Compound	Content (%)	References
Thymol	41.0–75.5	[107,109,110,111,112,113]
Carvacrol	2.2–77.6	[107,108,109,110,111,112,113]
β-myrcene	60.7	[112]
γ-terpinene	1.2–30.9	[107,109,110,111,112,113]
p-cymene	7.6–15.4	[107,109,110,112,113]
1,8-cineole	0.2–14.3	[109,110,113]
β-caryophyllene	0.4–13.4	[107,110,111,113]

**Table 7:** Prevailing components of *C. limon* essential oil.

Compound	Content (%)	References
Limonene	47.3–70.5	[121,123,124,125,126]
β-pinene	11.2–14.0	[123,124,125,126]
γ-terpinene	8.2–12.2	[121,123,124,125]
α-pinene	10.5	[126]
Geranial	0.7–4.5	[124,125,126]
β-laurene	4.2	[126]
Sabinene	0.8–3.4	[124,125]
Neral	3.2	[126]
Myrcene	1.4–2.7	[124,125]

## Data Availability

Data sharing is not applicable to this article.
